# Evaluation of PAC and FASP Performance: DIA-Based Quantitative Proteomic Analysis

**DOI:** 10.3390/ijms25105141

**Published:** 2024-05-09

**Authors:** Maria Stella Murfuni, Licia E. Prestagiacomo, Annarita Giuliano, Caterina Gabriele, Sara Signoretti, Giovanni Cuda, Marco Gaspari

**Affiliations:** Research Centre for Advanced Biochemistry and Molecular Biology, Department of Experimental and Clinical Medicine, Magna Graecia University of Catanzaro, 88100 Catanzaro, Italy; annarita.giuliano@studenti.unicz.it (A.G.); cgabriele86@gmail.com (C.G.); s.signoretti@unicz.it (S.S.); cuda@unicz.it (G.C.); gaspari@unicz.it (M.G.)

**Keywords:** benchmarking, protein aggregation capture, filter-aided sample preparation, Spectronaut, DIA-NN, MaxDIA

## Abstract

The aim of this study was to compare filter-aided sample preparation (FASP) and protein aggregation capture (PAC) starting from a three-species protein mix (*Human*, *Soybean* and *Pisum sativum*) and two different starting amounts (1 and 10 µg). Peptide mixtures were analyzed by data-independent acquisition (DIA) and raw files were processed by three commonly used software: Spectronaut, MaxDIA and DIA-NN. Overall, the highest number of proteins (mean value of 5491) were identified by PAC (10 µg), while the lowest number (4855) was identified by FASP (1 µg). The latter experiment displayed the worst performance in terms of both specificity (0.73) and precision (0.24). Other tested conditions showed better diagnostic accuracy, with specificity values of 0.95–0.99 and precision values between 0.61 and 0.86. In order to provide guidance on the data analysis pipeline, the accuracy diagnostic of three software was investigated: (i) the highest sensitivity was obtained with Spectronaut (median of 0.67) highlighting the ability of Spectronaut to quantify low-abundance proteins, (ii) the best precision value was obtained by MaxDIA (median of 0.84), but with a reduced number of identifications compared to Spectronaut and DIA-NN data, and (iii) the specificity values were similar (between 0.93 and 0.99). The data are available on ProteomeXchange with the identifier PXD044349.

## 1. Introduction

In mass spectrometry-based proteomics, sample processing encompasses several steps to extract, solubilize and digest the proteins [1]. The ideal proteomics protocol should be (i) compatible with different extraction buffers, (ii) suitable for low sample inputs and (iii) composed of a low number of steps and automatable in order to reduce the variability associated with the operator [2,3]. Though the choice of sample preparation protocol in mass spectrometry-based proteomics will largely depend on the goals of the study (e.g., the study of specific post-translational modifications or of specific subcellular compartments), a few approaches have been extensively adopted by proteomics laboratories and core facilities for whole-cell proteomic analysis. Among the most popular approaches which, at least partially, satisfy the characteristics listed above for the ideal proteomics protocol, it is worth mentioning filter-aided sample preparation (FASP) [4,5,6] and protein aggregation capture (PAC) [7]. The two protocols show considerable versatility even for challenging samples such as biological fluids, so evaluating their performance is of great interest.

FASP is a widely used protocol for proteomic sample preparation because it exhibits several strengths such as compatibility with different extraction buffers [8], the removal of all interferents prior to enzymatic digestion and, last but not least, the ability to concentrate diluted protein solutions on the filter unit, ensuring optimal conditions for the enzyme/substrate interaction. Given its potential, the protocol has been made amenable to automation [9], leading to two important advantages: increased throughput and reduced variability associated with the operator. Despite these promising features, the classical FASP protocol shows limitations with low-input samples since there appears to be an optimum ratio between the filter size and the amount of protein loaded. For this reason, a smaller filter size (Well-Plate μFASP) [10] has been used to improve the interaction between proteins and the filter surface for low-concentration samples, overcoming this limitation.

PAC, on the other hand, is a protocol based on magnetic beads. Proteins are precipitated on the beads by adding an organic solvent and subsequently digested. In detail, in order for proteins to settle on the surface of the beads, it is important (i) to use the correct bead–protein ratio, (ii) to operate at the optimal salt concentration and (iii) to remove cellular debris and nucleic acids which could interfere with the interaction. The lack of any one of these conditions inevitably leads to the loss of material [11]. Following the on-bead protein precipitation, all of the steps of the protocol are performed in a single test tube placed on the magnetic rack, which favors the separation of the bead/protein aggregate from the supernatants (lysis buffer plus washing). Repetitive washings of magnetic beads allow for the effective removal of detergents. Thus, after tryptic digestion, the peptide mixture is suitable for direct analysis by mass spectrometry without additional purification steps. Although the two protocols were developed to digest tens of micrograms of proteins, some modifications have allowed both protocols to be used for even smaller quantities. Nevertheless, specific methods are needed for the analysis of extremely small amounts of starting material. These have been developed by laboratories with expertise in low-input proteomics [12].

FASP and PAC sample preparation protocols along with the in-StageTip (iST) protocol developed by Mann and co-workers [13] were compared in a paper by Sielaff and colleagues [14]. The comparison was performed primarily on a qualitative basis (proteome coverage), as it did not include the use of mixed proteomes for probing diagnostic accuracy. Sielaff et al. evaluated the performance of the three protocols from the perspective of proteome coverage and quantitative precision (CVs) in the low-microgram range, achieving similar performance for the high-end of the interval (20 μg) but achieving better quantitative reproducibility in the case of SP3 and iST for the low-end of the tested range. In the work by Sielaff et al., MS detection was performed in UDMS^E^ mode, a specific type of data-independent acquisition (DIA). DIA analysis has become widely adopted in recent years, following great improvements in the sequential window acquisition mode and in data analysis software [15,16,17], in order to overcome the missing value problem derived by the stochastic nature of data-dependent acquisition (DDA) [18,19]. DIA is a sensitive method able to select and sequence even peptides deriving from low-abundance proteins. The main problem encountered with DIA data is the complexity of the spectra, since a single MS2 spectrum in a DIA file is not associated with a single peptide sequence, but it is the result of the fragmentation of multiple co-isolated precursors in a given *m*/*z* range. For this reason, while DIA overcomes the problem of missing values by providing a deep proteomic profile [20,21], it also generates very complex files. Thanks to the design of sophisticated software, however, today the analysis of DIA data is no longer a problem.

The aim of this study was to compare two widely adopted sample preparation protocols, FASP and PAC, in terms of proteome coverage and diagnostic accuracy using a three-species protein mix (*Human*, *Pisum sativum* and *Soybean*). Two different quantities of starting protein were processed by both protocols: a “routine” amount (10 µg) and a more challenging amount (1 µg). MS detection was performed in DIA mode. Since data analysis is a key step of every proteomics pipeline, especially in the case of DIA mode, raw data were processed using three of the most commonly used software: Spectronaut, MaxDIA [22] and DIA-NN [23]. Nevertheless, extensive benchmarking of DIA data analysis software was not the purpose of this study and can be found elsewhere [24].

## 2. Results and Discussion

The main objective of this study was to evaluate the diagnostic accuracy of two different digestion protocols combined with DIA analysis [24,25]. For this purpose, two different amounts (1 μg and 10 μg) of PmixA and PmixB were digested in quadruplicate. The repeatability of the two protocols was calculated through the Pearson coefficient (Figures S1 and S2), highlighting a good agreement between replicates across the entire dataset.

In our experience, FASP-derived peptide solutions might contain residual amounts of SDS or other detergents which might compromise LC performance in the long run; for this reason, we routinely perform StageTip SCX purification before LC-MS/MS analysis for FASP-digested samples. On the contrary, PAC-digested samples could, in principle, be injection-ready [26]. Considering that the micropurification step could add imprecision to the FASP method, thus favoring the PAC protocol, PAC samples were analyzed in two different modalities: (i) direct injection and (ii) injection after StageTip SCX purification. In this way, it was possible to assess both the advantage of the directed injection for PAC and the effect of the purification step on precision and proteome coverage. After sample processing, all 48 samples (16 FASP and 16 PAC-1 with purification, and 16 PAC-2 without purification) were analyzed by LC-MS/MS in DIA mode. DIA raw files were processed by Spectronaut, MaxDIA and DIA-NN. For the first two data analyses, we used a spectral library built by dividing 10 different fractions into a total of 20 μg of peptides (pool of PmixA and PmixB). The obtained results were investigated at two different levels: (i) qualitative analysis, to indicate the condition able to identify the highest number of proteins (Table S1), and (ii) quantitative analysis, to evaluate the diagnostic accuracy of the DIA method.

**Figure 1:**
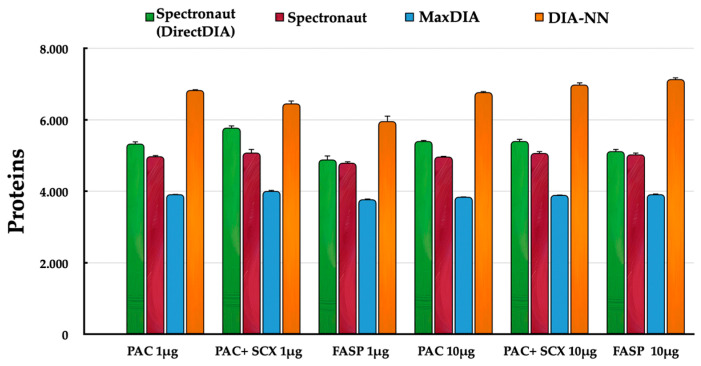
Identified proteins by FASP and PAC (1 μg and 10 μg in quadruplicate) using the three different software (Spectronaut, MaxDIA and DIA-NN).

From a qualitative point of view, the PAC-1 protocol (with purification) performed on 10 μg of starting material yielded the highest number of protein identifications (mean value of 5491), whereas the lowest number was obtained from the FASP protocol starting from 1 μg (mean value of 4855; Figure 1). Furthermore, although SCX purification adversely affects peptide recovery, in this experiment, the comparison between PAC-1 and PAC-2 showed a significant (*p* << 0.001) increase in the number of identified proteins (about 200 more proteins, on average; Table S2) regardless of the data analysis software used. Furthermore, the qualitative analysis enclosed the evaluation of missing values since even if the DIA method overcomes the stochastic nature of DDA, it does not completely solve the problem. As it can be seen in Figure S3, data completeness at the level of protein groups was higher than 94% in all conditions, suggesting that quantitative data would not have been substantially affected by the choice of the imputation strategy.

After outlining the main aspects of the qualitative data, our attention shifted to the quantitative data provided by FASP and PAC through the use of three different software. DIA data were analyzed by Spectronaut, MaxDIA and DIA-NN, and TPs, TNs, FPs and FNs were calculated (Table 1). Importantly, the very few proteins identified as regulated but showing a trend opposite to that expected (e.g., a soybean protein found downregulated in A) were removed from the TP count.

**Figure 2:**
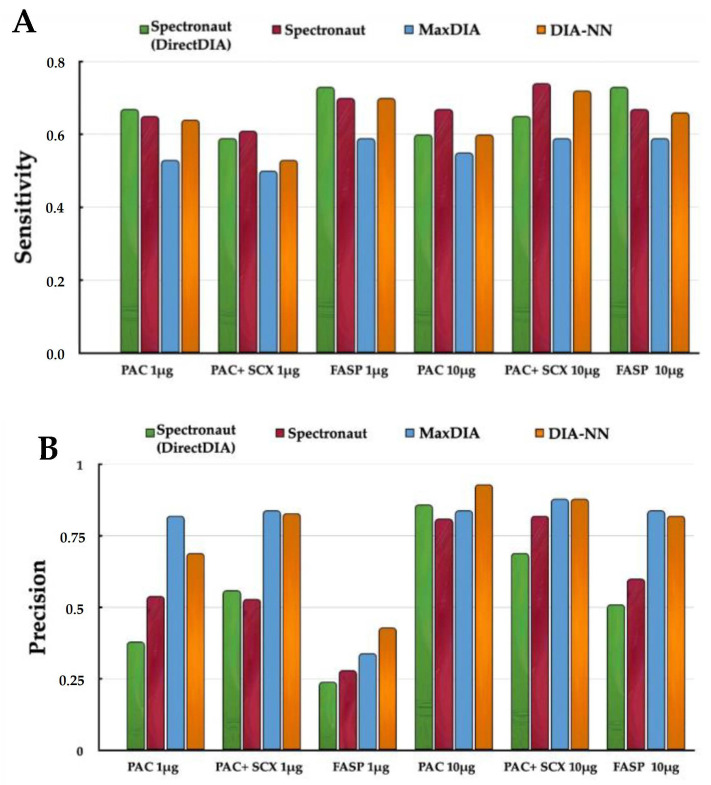
Sensitivity (**A**) and precision (**B**) obtained by FASP and PAC (1 μg and 10 μg in quadruplicate) using Spectronaut, MaxDIA and DIA-NN.

Regarding specificity and precision, the lowest average values were obtained for FASP 1 µg (0.84 and 0.32, respectively), while the highest values were obtained from PAC-1 10 µg (0.99 and 0.82, respectively) and PAC-2 10 µg (0.99 and 0.86; Figure 2 and Table S3).

**Figure 3:**
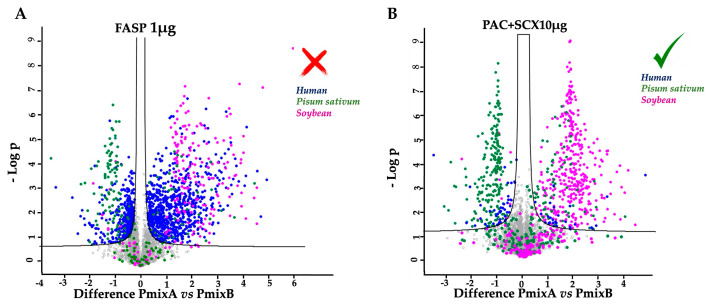
The figure encloses the volcano plot for the worst result (**A**) and for the best result obtained (**B**); the FDR threshold was set to 0.05.

Of all scenarios presented regarding sensitivity, the extremely low value found for FASP 1 µg was certainly noteworthy (Figure 3). This result was already predictable from the particularly high value of FNs.

After considering the accuracy of the method in relation to the experimental conditions, the performance of the three different software for data analysis was investigated. Overall, the specificity values returned by each software were similar, with the highest values equal to 0.99 obtained by MaxDIA and DIA-NN (median values across the six different binary comparisons made). However, there was one important aspect to consider: DIA-NN returned nearly twice as many identifications as MaxQuant. In addition, it also yielded a lower number of FPs. The same situation was not observed for sensitivity, where the best result (0.67) was obtained by processing the data with Spectronaut without the aid of a spectral library. In this case, Spectronaut showed its ability to better quantify low-abundance proteins (*Soybean* and *Pisum sativum*) compared to other software, thus detecting less FNs.

By observing the values shown in Table S3, it can be seen that the highest precision was obtained by MaxDIA (median precision equal to 0.84). Nevertheless, in the light of the lower number of identifications detected by MaxDIA, the obtained precision needed some consideration. Among the different software used, MaxDIA identified fewer proteins. Thus, it is reasonable to postulate that these were the most abundant proteins, i.e., the ones quantifiable with the lowest error, returning a good precision value. On the other hand, DIA-NN showed a compelling precision value (median of 0.83), especially when considering that it quantified a much higher number of low-abundance proteins, achieving a deeper proteome coverage with respect to MaxDIA.

All other values, especially those obtained from Spectronaut (0.54 for Direct-DIA and 0.57 for the analysis with the spectral library), were unacceptable values for a quantitative analysis. Since Spectronaut showed very high sensitivity, in order to reduce false positives and improve precision, a new statistical analysis was performed on the best condition only (PAC-1 10 µg). In detail, without modifying any parameter in the Spectronaut software, the matrix with the quantified proteins was analyzed in Perseus using an FDR at 0.01 (Table 2). As reported in the table, this strategy certainly led to the reduction in the absolute number of regulated proteins but provided a more accurate list of candidates, with the precision improved from 0.82 to 0.94. Regarding the sensitivity, the decrease from 0.75 to 0.56 is related to the increase in FNs.

Finally, to further investigate our results, it was verified if the accuracy of the method could be improved by quantifying the proteins with a minimum of two peptides. The aim of this new analysis was to understand whether improving the data quality (more accurate measurements with more peptides) improved the accuracy of the quantitative method. In the light of the considerations, for the software that allowed us to modify the quantitative strategy (Spectronaut and MaxDIA), the number of TPs, TNs, FPs and FNs were calculated (Table 3). By performing a comparison between the two analyses (minimum of one peptide vs minimum of two peptides), Spectronaut showed small variations both for the specificity and precision (Table S4), though sensitivity generally improved due the decrease in the FN rate.

Compared to Spectronaut, MaxDIA showed a similar value for specificity but a reduced sensitivity (0.69); moreover, a worsening of precision for small quantities was observed (1 µg).

## 3. Materials and Methods

All chemicals used in the experiments here described were purchased from Sigma-Aldrich (St. Louis, MO, USA) unless otherwise indicated.

### 3.1. Sample Preparation

HEK 293 was lysed with RIPA buffer and the protein concentration amount was estimated by using the Bradford Protein Assay. In detail, a volume of 200 µL of HEK 293 cell lysate with a concentration equal to 19 μg/µL was centrifugated to discard the pellet. A volume of 105 µL of the lysate (2 mg of protein) was brought to 400 μL with 100 mM Tris buffer at pH 8.0 and 1% SDS (*v/v*) obtaining an approximate concentration of 5 μg/µL. *Pisum sativum* and *Soybean* powders (5 mg) were dissolved in 2 mL of the same buffer and vortexed (approximate concentration equal to 2 μg/µL); a total of 400 μL was used for each proteome.

To reduce and alkylate disulfide bonds, 40 μL of 100 mM dithiothreitol (DTT) was added to 400 μL of each solution, followed by the addition of 48 μL of 200 mM of iodoacetamide (IAA); each step included 1 h of incubation at 37 °C with shaking (650 rpm on a Thermomixer). Finally, 8 μL of 100 mM DTT was added to quench residual iodoacetamide and the incubation was allowed to proceed at 37 °C for 30 min.

### 3.2. Preliminary Protein Quantification

Before creating the protein mixtures, approximately 10 μg of reduced and alkylated proteins was digested by using the PAC protocol [27] (explained in detail below) in order to estimate the protein amount of each stock solution (*Human, Soybean* and *Pisum sativum*); the estimated ratio of trypsin to protein was 1 to 50 (Sigma-Aldrich, product no. T6567). After tryptic digestion, the peptide solutions were 100 μL; from each solution, a 20 μL aliquot was withdrawn, combined with 30 μL of solution A (0.1% formic acid (FA) and 2% of acetonitrile; ACN) and analyzed by LC-MS/MS analysis.

To estimate the protein quantity, a calibration line was constructed by preparing four different solutions (5, 10, 25 and 50 ng/μL) of a HeLa digest stock (100 ng/μL, Thermo Fisher Scientific); 2 μL of each solution was analyzed by LC-MS/MS. For all samples, the same acquisition method was used. Using the HeLa samples as external standards, an estimation of the protein concentration of the three protein mixtures was obtained by interpolation using, as a measurement, the log10 of the area under the curve (AUC) calculated from the LC-MS/MS files. The following approximate concentrations were obtained: (i) *Human* proteins equal to 1.6 μg/μL, (ii) *Soybean* equal to 100 ng/μL and (iii) *Pisum sativum* equal to 640 ng/μL.

### 3.3. Protein Blend Preparation

After protein estimation, the three proteomes (*Human*, *Soybean* and *Pisum sativum*) were mixed in order to create two different blends, as shown in Table 4. The two blends created a protein fold-change ratio (PmixA/PmixB) of exactly 1 for *Human* proteins, 3 for *Soybean* and 0.5 for *Pisum sativum* proteins.

### 3.4. Protein Digestion

In order to compare the performance of the FASP and PAC protocols, two different amounts of proteins (1 and 10 μg) for Pmix A and Pmix B were digested in quadruplicate, thus processing a total of 32 samples.

#### 3.4.1. FASP Protocol

Before being loaded into the filter, the mixtures of reduced and alkylated proteins were prepared, as reported in Table 5. These dilutions allowed us to load the same volume of protein solution for each sample, and thus to perform all FASP digestions in parallel in the same batch.

**Figure 4:**
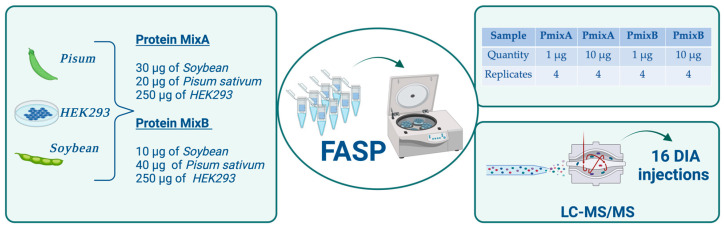
The figure summarizes the information relative to the FASP protocol. Created with BioRender.com.

Since each condition was performed in quadruplicate, overall, 16 samples were digested by using the FASP protocol (Figure 4). Except for the step of cysteine alkylation, which in this case was performed in solution, the FASP protocol was carried out as previously reported [28]. Protein reduction and alkylation outside the filter did have a negligible effect in terms of proteome coverage (data not shown) but allowed us to minimize potential differences and biases between the two protocols. Protein digestion was performed by adding 200 ng of trypsin to both the 1 μg and the 10 μg mixes. The choice of adding the same trypsin amount to both mixes was dictated by the fact that increasing the enzyme/substrate ratio is beneficial in cases of lower substrate concentrations [29].

#### 3.4.2. PAC Protocol

The PAC protocol [27] requires the right proportions between beads, protein amounts and aqueous and organic solvents. In our laboratory procedure, the protein solution starting volume was 20 μL. For this reason, samples were diluted as reported in Table 6.

**Figure 5:**
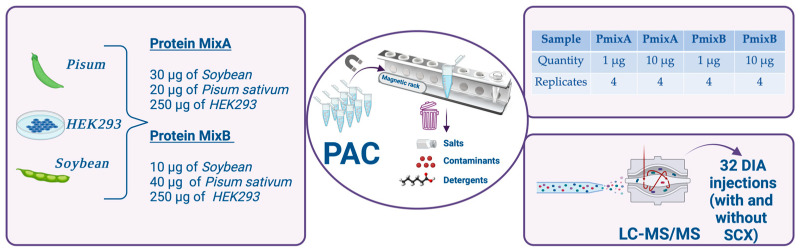
The figure shows the information about the PAC protocol. Created with BioRender.com.

Proteins were digested as follows. A total of 5 μL of MagReSyn Hydroxyl beads (100 μg of beads, Resyn Bioscicences), previously conditioned with 70% ACN (*v*/*v*), was added to all samples. Protein precipitation on the beads was promoted by bringing the ACN concentration to 70% and subsequently incubating the suspension in a thermomixer at room temperature with stirring at 1100 rpm for ten minutes. The samples were then placed in a magnetic rack and subjected to several washing steps (all performed on the rack): three washes with 100% of ACN and one wash with 70% ethanol. The last supernatant was removed; then, 51 μL of digestion buffer (50 mM triethylammonium bicarbonate, TEAB, containing 200 ng of trypsin) was added to the beads. The suspension was incubated overnight at 37 °C and 1100 rpm (Figure 5).

### 3.5. SCX Purification

In our experience, the peptide mixture obtained after FASP digestion may contain residual amounts of detergent, and for this reason, all samples processed by the FASP protocol were purified through strong cation exchange (SCX) purification [30]. PAC samples, on the other hand, allow for direct injection (without a purification step), but to perform an equal comparison, PAC digests were analyzed both directly (without purification) and after SCX purification.

In detail, half of the eluate from the FASP and PAC protocols was purified by SCX StageTips for the removal of traces of residual detergent. In order to prepare the StageTips, a piece of Empore^TM^-3M SCX (Millipore) resin was withdrawn using a blunt-ended syringe needle (gauge 16). For StageTip purification, 100 μL of FASP and 25 μL of PAC eluates were diluted 4-fold in wash solution 2 (0.5% formic acid (FA) and 80% ACN); since salts interfere with the binding of peptides to the SCX stationary phase, this dilution step was critical to reduce the salt concentration below 5 mM. Diluted samples were loaded on the StageTips and washed with 50 μL of wash solution 1 (0.5% FA and 20% ACN) and 50 μL of wash solution 2. Peptides were then eluted in 10 μL of 500 mM ammonium acetate containing 20% ACN and dried at 30 °C in a speed-vac; the samples with the starting protein amount of 1 μg (theoretical 500 ng of purified peptides) were resuspended in 25 μL of solution A, while the samples with the starting protein amount of 10 μg (theoretical 5 μg of purified peptides) were resuspend in 100 μL of solution A.

### 3.6. High pH Reversed-Phase C_18_ Fractionation

To build the DIA library, 20 μg of total peptides (PmixA and PmixB) was loaded on a high-capacity StageTip containing a greater amount of C18 stationary phase (threefold) to perform basic reversed-phase fractionation, as previously reported [31]. In detail, the peptide mixture was acidified with 0.1% of trifluoroacetic acid (TFA) in order to achieve a pH lower than 3. Before diluted sample loading, the stationary phase was washed and conditioned with 50 μL of solution A (0.1% TFA and 50% ACN) and 50 μL of solution B (0.1% TFA), respectively. The stationary phase was washed with solution B after sample loading, and finally, the peptides were fractionated using solutions composed by 10 mM TEAB, 0.2% ammonium hydroxide and increasing concentrations of ACN (4, 8, 12, 16, 20, 24, 28, 32, 40 and 80%).

Each fraction was eluted in 30 μL and dried at 30 °C in a speed-vac; the fractions were resuspended in 40 μL of solution A.

Ten fractions were analyzed in DDA mode, and the identified peptides were used to draw up the DIA spectral library.

### 3.7. LC-MS/MS Analysis

Peptides were separated by using an Easy nLC-1000 chromatographic instrument coupled to an Exploris 480 mass spectrometer (both from Thermo Scientific, Bremen, Germany).

For the generation of the spectral library, 2 μL from each basic RP fraction was separated using a linear gradient of 63 min at a flow rate of 300 nL/min on a 15 cm, 75 μm i.d. column, in-house packed with 3 μm C18 silica particles (Dr. Maisch). The gradient was generated using mobile phase A (0.1% FA and 2% ACN) and mobile phase B (0.1% FA and 80% ACN). Peptide separation was achieved at a flow rate of 300 nL/min using the following gradient: from 4% B to 12% B in 16 min, from 12% B to 36% B in 16 min and from 36% B to 100% B in 8 min; the column was cleaned for 5 min with 100% B. For the analysis of fractions, the mass spectrometer operated in DDA mode using a top-12 method. In detail, the MS full scan was 375–1400 *m*/*z* with a resolution of 60,000, AGC target of 1 × 10^6^ and maximum injection time of 50 ms. The mass window for the isolation of the precursor was 1.6 *m*/*z*, with a resolution of 30,000, an AGC target of 1 × 10^6^ and a maximum injection time “custom”; HCD fragmentation was set at normalized collision energy of 30 and dynamic exclusion of 10 s.

For the analysis of FASP and PAC individual samples, 100 ng of peptide mixtures was separated using the same gradient described above, while the mass spectrometer operated in DIA mode. The DIA method was composed of 32 consecutive MS2 windows acquired at 30,000 resolution, with an AGC target of 5 × 10^5^ and a maximum injection time of 50 ms. In details, the DIA method enclosed 24 windows with an isolation window of 15 *m*/*z*, 5 windows with an isolation of 30 *m*/*z* and 3 windows with an isolation window of 50 *m*/*z*; the overlap for each window was equal to 0.5 *m*/*z*. The resulting *m*/*z* range was from 350 to 1000.

### 3.8. DIA Data Processing

The raw files were analyzed using three different software: MaxQuant (version 2.1.3.0, Max-Planck-Institute of Biochemistry 2021), Spectronaut (version 18.4, Biognosys AG, Switzerland) and DIA-NN (version 1.8.1). DIA analysis in MaxQuant and Spectronaut software was performed using our experimental spectral library, while DIA-NN was run in library-free mode by performing deep learning-based library generation. In addition, Spectronaut analysis was also performed using the Direct-DIA mode.

The following databases were used for all analyses: *Human* (79,684 sequences downloaded on 30 May 2022), *Pisum sativum* (64,176 sequences downloaded on 18 October 2023) and *Soybean* (74,863 sequences downloaded on 18 October 2023).

#### 3.8.1. MaxQuant

Raw files of the ten fractions were imported in MaxQuant (version 2.1.3.0) to create our spectral library using the MaxQuant algorithm; this process provided three different files: msms.txt, evidence.txt and peptides.txt. The used parameters were as follows: protein databases (*Human*, *Pisum sativum* and *Soybean*), first and main search peptide tolerance, respectively, of 20 ppm and 4.5 ppm, trypsin/P as an enzyme, and two missed cleavages. Carbamidomethylation of cysteines was set as static modification, and oxidation of methionine and protein N-terminal acetylation were allowed as variable modifications. The value of FDR was set to 0.01 and only the peptides with >7 amino acid residues were selected for identification; only unique ones were used for protein quantification.

DIA runs were analyzed by the MaxDIA algorithm by loading the three spectral library files (msms.txt, evidence.txt and peptides.txt), generated from the DDA experiments, in the appropriate tab; the other settings were the same as reported above for library processing.

#### 3.8.2. Spectronaut

In order to build the spectral library, raw files from high-pH reversed-phase fractionation were analyzed in Spectronaut (version 18.4), setting the Q-value cut-off to 0.01, with a minimum and maximum of fragment ions of 3 and 6, respectively.

DIA runs were loaded in Spectronaut, and the obtained identifications were filtered by a Q-value of 0.01. Protein quantification was performed using “Only Protein Group Specific” and missing values in the precursor identified in at least 35% of runs were imputed using global imputing. For protein quantification in the panel “major grouping”, the minimum number of peptides were set to 1 and the maximum to 3. Runs were normalized through global normalization.

DIA files were also analyzed in Direct-DIA mode, thus without spectral library, using the same settings for quantitative analysis. Direct-DIA was performed by “DirectDIA+Fast”.

#### 3.8.3. DIA-NN

Protein sequences of the three proteomes were imported in DIA-NN and a spectral library was predicted using the deep learning algorithms implemented in DIA-NN (version 1.8.1). The used parameters were as follows: trypsin as an enzyme, allowing for up to one missed cleavage site, charge states of 1–4 for peptides consisting of 7–40 amino acids, carbamidomethylation of cysteines, oxidation of methionine and an FDR of 1% for precursor identifications; the quantification strategy was set to “Any LC, high accuracy”, whereas normalization was set to global. Matching between runs was activated.

### 3.9. Statistical Analysis

To perform statistical analysis, the matrices obtained from the different software were imported in Perseus (version 2.0.6.0, Max-Planck-Gesellschaft, München). In detail, protein intensity values were transformed in the logarithmic scale (log2); only proteins quantified in at least three replicates of at least one sample group (PmixA or PmixB) were kept, while missing values were imputed using default settings (width of 0.3 SD; down shift of 1.8 SD).

Significantly different proteins between PmixA and PmixB were detected by Student’s *t*-test corrected for multiple hypothesis testing with a Permutation-based FDR equal to 0.05. An S0 value of 0.2 was used. Histograms were generated by using Numbers (v 13.2).

### 3.10. Diagnostic Accuracy of the Method

Sensitivity, specificity and precision were calculated for each tested condition to evaluate the diagnostic accuracy of the method. Since the composition of the protein mix was known, it was possible to evaluate the following parameters: (i) false positives (FPs), i.e., the *Human* proteins found to be regulated; (ii) true negatives (TNs), i.e., the *Human* proteins classified as unchanged; (iii) true positives (TPs), i.e., the *Soybean* and *Pisum sativum* proteins found to be regulated; (iv) false negatives (FNs), i.e., *Soybean* and *Pisum sativum* proteins classified as unchanged. All of the proteins assigned to more than one proteome were excluded.

Based on these parameters, the sensitivity, specificity and precision of our method were calculated through these ratios:

(i) Sensitivity: TP/(TP + FN);
(ii) Specificity: TN/(TN + FP);
(iii) Precision: TP/(TP + FP).

## 4. Conclusions

Through benchmarking experiments, the diagnostic accuracy of two widely used sample preparation protocols was evaluated, also considering the data analysis pipeline.

Our data indicated that the regular FASP protocol, with respect to PAC, showed a lower number of identifications and worse diagnostic accuracy for low quantities. SCX purification after PAC digestion had no negative effects on either proteome coverage or diagnostic accuracy. However, direct injection into the LC-MS system after PAC digestion represents an important advantage over FASP and, for this reason, should be considered as the preferred route.

Regarding the software, the obtained values for specificity indicated that Spectronaut, MaxDIA and DIA-NN showed a similar ability in quantifying high-abundance proteins. Evaluating the values obtained for precision and sensitivity, instead, it was clear that each software returned its own interpretation of low-abundance proteins. Currently, in fact, the integration of the signal deriving from low-abundance proteins represents the real weak point of data analysis in MS-based proteomics, thus marking the need to improve and frequently update the bioinformatic support to accurately interpret the sophisticated mass spectrometer data.

Though extensive benchmarking of DIA data analysis software was not the purpose of this study, and though software performance may dramatically change following software updates, we found that DIA-NN delivered good overall performance in terms of proteome coverage and precision.

## Figures and Tables

**Table 1:** Total identified proteins for *Human*, *Pisum sativum* and *Soybean*. TPs (*Pisum sativum* and *Soybean* proteins found changed), TNs (*Human* proteins found unchanged), FPs (*Human* proteins found changed), and FNs (*Pisum sativum* and *Soybean* proteins found unchanged) are also reported.

	Condition	*Human*	*Pisum sativum*	*Soybean*	TP	TN	FP	FN
**Spectronaut** **Direct-DIA**	PAC-1 µg	3799	184	287	315	3293	506	156
	PAC+SCX-1 µg	4176	217	287	395	3942	234	209
	FASP-1 µg	3550	170	242	302	2583	967	110
	PAC-10 µg	3879	198	286	289	3831	48	195
	PAC+SCX-10 µg	4141	232	307	352	4085	56	187
	FASP-10 µg	3718	178	239	303	3423	295	114
**Spectronaut**	PAC-1 µg	3651	141	205	226	3457	194	120
	PAC+SCX-1 µg	3748	142	203	211	3562	186	134
	FASP-1 µg	3511	137	194	231	2913	598	100
	PAC-10 µg	3655	144	204	234	3601	54	114
	PAC+SCX-10 µg	3741	145	203	257	3685	56	91
	FASP-10 µg	3716	142	205	233	3558	158	114
**MaxDIA**	PAC-1 µg	3388	99	172	144	3357	31	127
	PAC+SCX-1 µg	3488	103	169	136	3463	25	136
	FASP-1 µg	3324	97	162	154	3031	293	105
	PAC-10 µg	3302	96	167	145	3292	10	118
	PAC+SCX-10 µg	3353	103	173	163	3330	23	113
	FASP-10 µg	3451	100	171	161	3421	30	110
**DIA-NN**	PAC-1 µg	6076	298	409	476	5878	198	231
	PAC+SCX-1 µg	6248	329	400	405	6166	82	324
	FASP-1 µg	5413	252	319	429	4896	517	142
	PAC-10 µg	6032	313	417	455	5997	35	275
	PAC+SCX-10 µg	6309	325	437	570	6230	76	192
	FASP-10 µg	5821	297	337	433	5727	94	201

**Table 2:** The table reports the values obtained by applying different FDR thresholds (0.05 and 0.01) in Perseus to the Spectronaut matrix. This strategy was applied only to the best condition (PAC-1 10 µg).

	Condition	*Human*	*Pisum sativum*	*Soybean*	TP	TN	FP	FN
**Spectronaut**	FDR 0.05	3741	145	203	257	3685	56	91
	FDR 0.01	3741	145	203	196	3728	13	152

**Table 3:** The table shows the number of TPs, TNs, FPs and FNs calculated starting from a matrix composed of proteins quantified by a minimum of 2 peptides.

	Condition	*Human*	*Pisum sativum*	*Soybean*	TP	TN	FP	FN
**Spectronaut** **Direct-DIA**	PAC-1 µg	2533	81	103	149	2264	269	35
	PAC+SCX-1 µg	2741	98	112	147	2600	141	63
	FASP-1 µg	2172	71	86	146	1661	511	11
	PAC-10 µg	2587	88	109	163	2571	16	34
	PAC+SCX-10 µg	2773	98	122	189	2744	29	31
	FASP-10 µg	2410	81	93	151	2248	162	23
**Spectronaut**	PAC-1 µg	2250	62	82	112	2135	115	32
	PAC+SCX-1 µg	2257	62	81	103	2148	109	40
	FASP-1 µg	2107	59	73	106	1746	361	26
	PAC-10 µg	2213	59	80	118	2184	29	21
	PAC+SCX-10 µg	2252	63	79	123	2224	28	19
	FASP-10 µg	2229	59	81	121	2139	90	19
**MaxDIA**	PAC-1 µg	2990	82	130	144	2925	65	68
	PAC+SCX-1 µg	3055	82	126	135	3024	31	73
	FASP-1 µg	2888	80	121	143	2544	344	58
	PAC-10 µg	2947	78	118	131	2940	7	65
	PAC+SCX-10 µg	2973	80	130	148	2957	16	62
	FASP-10 µg	3001	80	128	147	2983	18	61

**Table 4:** PmixA and PmixB composition.

Pmix	Volume of *Human*	Volume of *Soybean*	Volume of *Pisum sativum*	Final Concentration
A	156 μL (250 μg)	300 μL (30 μg)	32 μL (20 μg)	615 ng/μL
B	156 μL (250 μg)	100 μL (10 μg)	62.5 μL (40 μg)	942 ng/μL

**Table 5:** Dilution of protein mixes before FASP digestion.

Pmix	Pickup Volume	Added Volume of 100 mM Tris HCl at pH 8.0	Loaded Volume on the Filter	Loaded Protein Amount on the Filter
A	10 μL	790 μL	150 μL	1 μg
A	95 μL	755 μL	150 μL	10 μg
B	6 μL	794 μL	150 μL	1 μg
B	60.5 μL	789.5 μL	150 μL	10 μg

**Table 6:** Dilution of protein mixes before PAC digestion.

Pmix	Pickup Volume	Added Volume of TEAB 50 mM	Loaded Volume on the Beads	Loaded Protein Amount on the Filter
A	8.5 μL	91.5 μL	20 μL	1 μg
A	82 μL	18 μL	20 μL	10 μg
B	5.5 μL	94.5 μL	20 μL	1 μg
B	55 μL	45 μL	20 μL	10 μg

## Data Availability

The mass spectrometry proteomics data have been deposited in the ProteomeXchange Consortium (http://proteomecentral.proteomexchange.org) via PRIDE partner repository with the dataset identifier PXD044349. Reviewer account details: (i) reviewer_pxd044349@ebi.ac.uk (username) and (ii) cI1g0Yjw (password).

## Supplementary Materials

The following supporting information can be downloaded at: https://www.mdpi.com/article/10.3390/ijms25105141/s1.
