# Supplementary material for: Evaluation of PAC and FASP Performance: DIA-Based Quantitative Proteomic Analysis
Source: Int J Mol Sci. 2024 May 9;25(10):5141. doi: 10.3390/ijms25105141 (PMC11121386; doi:10.3390/ijms25105141)
Supplement: Supplementary file 1 [file ijms-25-05141-s001.zip › Supplementary Figures.pdf]

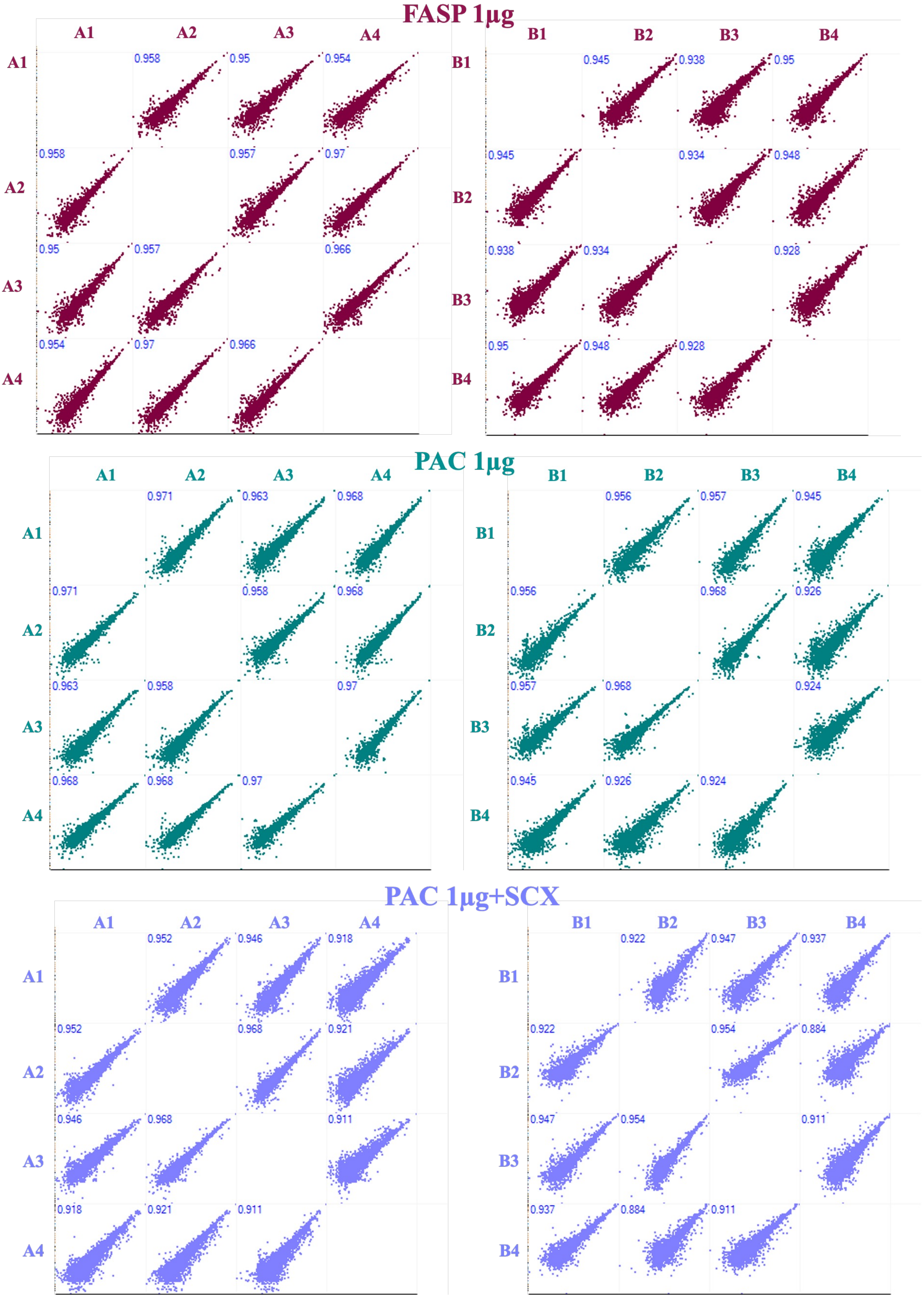

Figure S1: Pearson correlation coefficients between the replicates of 1 µg.

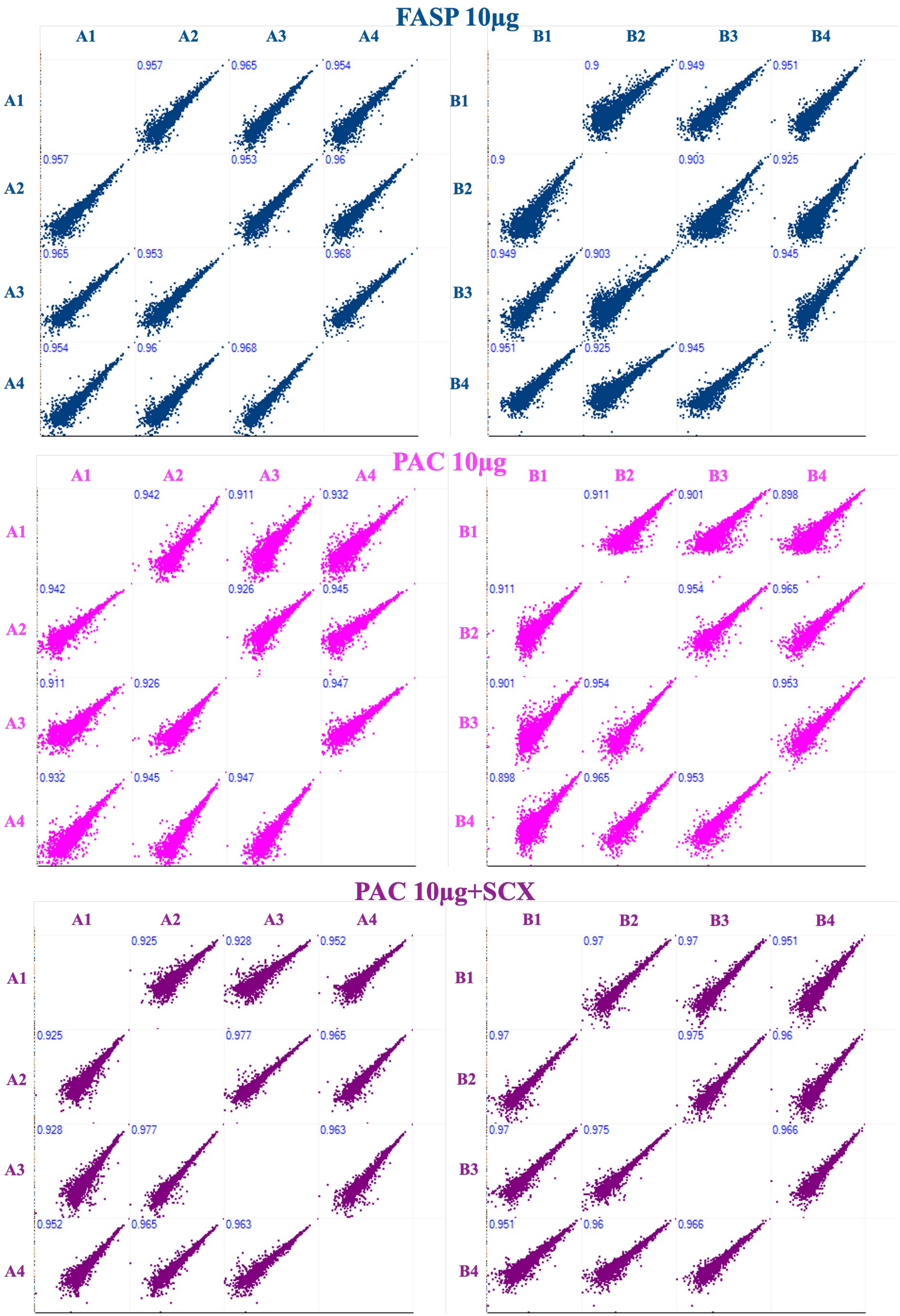

Figure S2: Pearson correlation coefficients between the replicates of 10 μg.

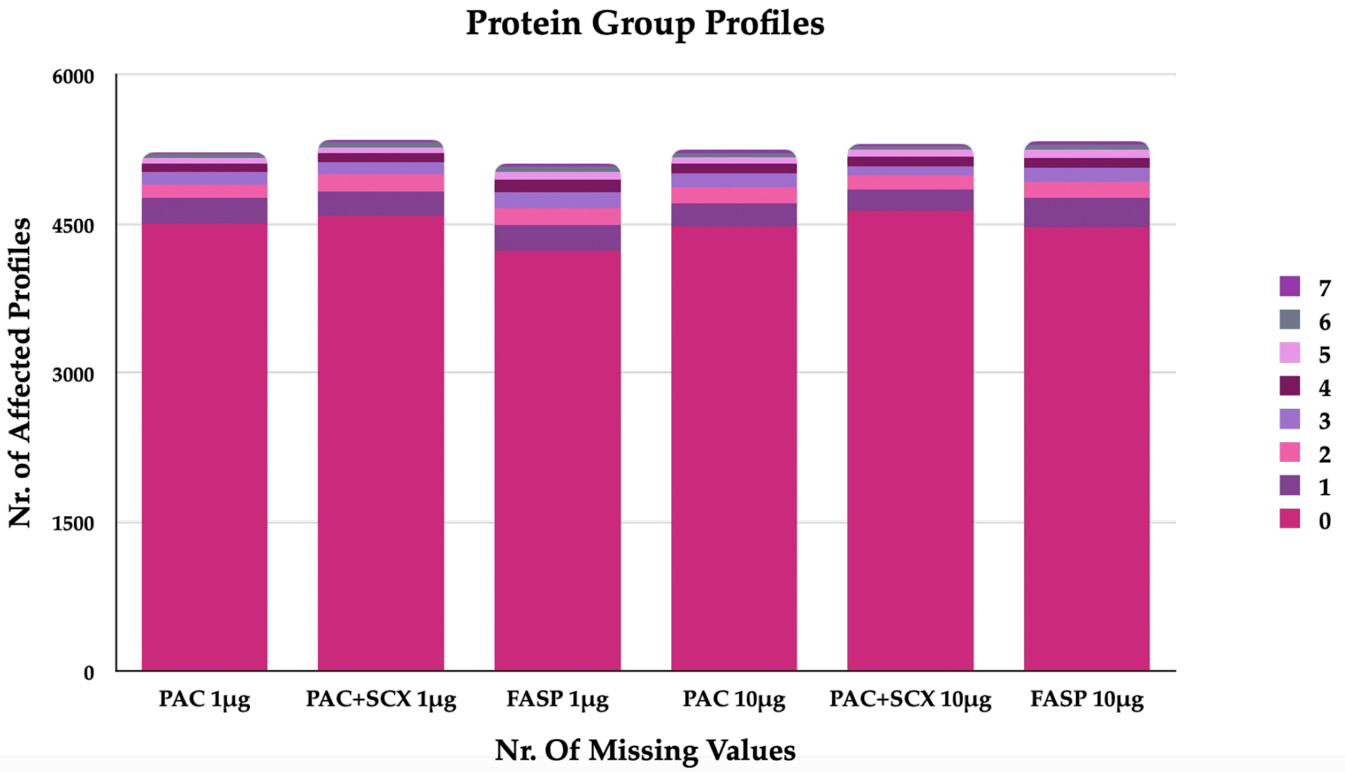

Figure S3: Missing values at the protein groups level.
